# ATTED-II in 2014: Evaluation of Gene Coexpression in Agriculturally Important Plants

**DOI:** 10.1093/pcp/pct178

**Published:** 2014-01-10

**Authors:** Takeshi Obayashi, Yasunobu Okamura, Satoshi Ito, Shu Tadaka, Yuichi Aoki, Matsuyuki Shirota, Kengo Kinoshita

**Affiliations:** ^1^Graduate School of Information Sciences, Tohoku University, 6-3-09, Aramaki-Aza-Aoba, Aoba-ku, Sendai, 980-8679 Japan; ^2^Core Research for Evolutional Science and Technology (CREST), Japan Science and Technology Agency, Kawaguchi, Saitama, Japan; ^3^Graduate School of Engineering, Tohoku University, 6-6-04, Aramaki-Aza-Aoba, Aoba-ku, Sendai, 980-8579 Japan; ^4^Institute of Development, Aging, and Cancer, Tohoku University, Sendai, 980-8575 Japan; ^5^Tohoku Medical Megabank Organization, Tohoku University, Sendai, 980-8573 Japan

**Keywords:** Arabidopsis, Comparative transcriptomics, Database, Gene coexpression, Gene network, Non-model species

## Abstract

ATTED-II (http://atted.jp) is a database of coexpressed genes that was originally developed to identify functionally related genes in Arabidopsis and rice. Herein, we describe an updated version of ATTED-II, which expands this resource to include additional agriculturally important plants. To improve the quality of the coexpression data for Arabidopsis and rice, we included more gene expression data from microarray and RNA sequencing studies. The RNA sequencing-based coexpression data now cover 94% of the Arabidopsis protein-encoding genes, representing a substantial increase from previously available microarray-based coexpression data (76% coverage). We also generated coexpression data for four dicots (soybean, poplar, grape and alfalfa) and one monocot (maize). As both the quantity and quality of expression data for the non-model species are generally poorer than for the model species, we verified coexpression data associated with these new species using multiple methods. First, the overall performance of the coexpression data was evaluated using gene ontology annotations and the coincidence of a genomic feature. Secondly, the reliability of each guide gene was determined by comparing coexpressed gene lists between platforms. With the expanded and newly evaluated coexpression data, ATTED-II represents an important resource for identifying functionally related genes in agriculturally important plants.

## Introduction

Recent high-throughput sequencing technologies have made it possible to generate genomic and transcriptomic data for non-model species. Annotation of these new sequences is typically accomplished by comparison with annotations of known orthologs. However, in contrast to clear orthologous relationships that characterize animal genomes, these types of relationships can be quite complicated in plants because of gene duplication events (Tang et al. 2008). Gene expression patterns can help address this problem, i.e. distinguish between paralogous genes, by providing clues concerning their biological roles. Genes involved in related biological pathways are generally expressed together, and thus, information about gene coexpression is key to understanding biological systems at the molecular level. Coexpression data have been used in many different experimental designs, including gene targeting, regulatory investigations and identifying protein–protein interactions (Aoki et al. 2007, Usadel et al. 2009, Obayashi and Kinoshita 2010).

We have constructed ATTED-II, which is a database of coexpressed genes for Arabidopsis (Obayashi et al. 2007), and have continuously improved it to increase its functionality, e.g. by incorporating condition-specific coexpression and the ability to draw networks (Obayashi et al. 2009, Obayashi et al. 2011). These tools can help identify functional gene relationships, so that reverse genetics and molecular biological techniques can be used to confirm predicted gene functions (Obayashi and Kinoshita 2010).

A grand challenge of plant science is to take the knowledge gained from model species (Arabidopsis and rice, in particular) and apply it to non-model species, other crops and trees (Godfray et al. 2010). To address this issue, we have expanded ATTED-II to include four dicots (soybean, poplar, grape and alfalfa) and one monocot (maize), which will facilitate the analysis of gene coexpression in non-model species while maintaining the reliability of the original coexpression indexes. For Arabidopsis, we prepared RNA sequencing (RNAseq)-based coexpression data and refined the microarray-based data. Although several databases, including the previous version of ATTED-II, provide coexpression data for multiple plant species (Toufighi et al. 2005, Mutwil et al. 2008, Jupiter et al. 2009, Ogata et al. 2010, Hamada et al. 2011, Mutwil et al. 2011, Patel et al. 2012, Yim et al. 2013), the quality of the data is not fully evaluated. Compared with the Arabidopsis data, the quality of coexpression data for other organisms is quite poor, primarily because of the limited number of microarray experiments. We have previously used gene ontology (GO) annotations to assess the accuracy of coexpression data (Obayashi and Kinoshita 2009, Kinoshita and Obayashi 2009), but GO annotations for non-model species are also less accurate than are those for model species. Thus, this approach did not work reliably.

To overcome this deficiency, we measured the degree of coincidence between coexpression data and a ‘genomic feature’, e.g. codon usage. Because genomic features are available for every gene, the quality of this type of information is consistent between species. Codon usage is a genomic feature related to coexpression (Plotkin et al. 2004, Najafabadi et al. 2009). We measured the degree of coincidence between coexpression and similarities in codon usage. The overall coincidence score seems to be a good measure of the quality of the coexpression data. In addition to an assessment of the overall performance of the gene coexpression data set, we also evaluated each coexpressed gene pair. This was accomplished by comparing coexpressed gene lists between platforms. If coexpression of two genes is conserved in two or more species, the reliability of that relationship is greatly enhanced, and the likelihood that experimental or technical artifacts are present is reduced (Stuart et al. 2003, Oti et al. 2008, Movahedi et al. 2011, Obayashi and Kinoshita 2011).

By filtering out less reliable gene coexpression data, the remaining data can be applied to non-model species with a greater degree of confidence. With the new coexpression data and added performance evaluations, the improved ATTED-II is a powerful database for identifying functionally related genes in agriculturally important plants.

## Results and Discussion

### New coexpression data for seven species

**Fig. 1:**
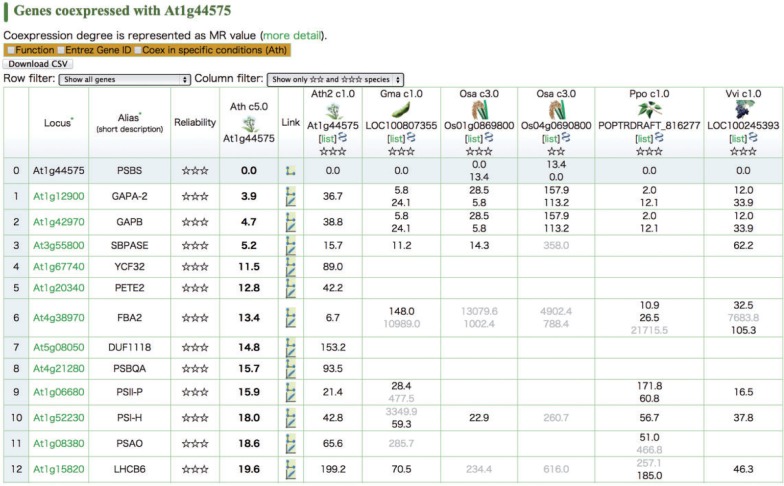
An example of a coexpressed gene list in ATTED-II. The Arabidopsis PSBS gene is used as the example of a guide gene, and coexpressed genes are shown along with their mutul rank (MR) values (a smaller MR value indicates a stronger coexpression). The six columns on the right indicate the degree of coexpression for ortholog pairs in other species (or another Arabidopsis platform). Coexpression with an MR value >200 is considered weak (gray text). A blank cell means that coexpression data were not available. The reliability was calculated on the basis of coexpression conservation and is represented by stars. Three stars indicate excellent reliability, whereas no stars indicates not reliable. This list is available at http://atted.jp/cgi-bin/coex_list.cgi?gene=At1g44575.

We first updated the coexpression data sets for Arabidopsis and rice by downloading microarray data from ArrayExpress (Rustici et al. 2013), which increased the number of Arabidopsis (*Arabidopsis thaliana*) microarrays from 1,388 to 11,171 and the number of rice (*Oryza sativa*) microarrays from 130 to 1,214. We also prepared new coexpression data sets for soybean (*Glycine max*), poplar (*Populus* sp.), grape (*Vitis Vinifera*), alfalfa (*Medicago truncatula*) and maize (*Zea mays*). In addition to the microarray-based coexpression data, we acquired RNAseq-based coexpression data for Arabidopsis. This helped resolve microarray-specific problems, especially for poorly expressed genes. Although the number of experiments in the RNAseq version (Ath2.c1-0) is currently limited, we anticipate that this will be a short-term problem. One prominent characteristic of the RNAseq data is deep coverage. Almost all Arabidopsis genes are included (Ath2.c1-0, 94% of the protein-encoding genes), representing a significant advantage over the microarray-based coexpression data set (Ath.c5-0, 76% of the protein-encoding genes) (Table 1). RNAseq and microarray coexpression data sets can now be viewed at the same time (Fig. 1).

**Table 1:** Coexpression data in ATTED-II version 7.1

Species	Version	No. of genes	Gene coverage (%)*^a^*	No. of experiments	No. of samples*^b^*	Platform	Release date
Arabidopsis	Ath.c5-0	20,836	76	737	11,171	A-AFFY-2	May 23, 2013
Arabidopsis	Ath2.c1-0	25,838	94	28	328	RNAseq	August 17, 2013
Soybean	Gma.c1-0	15,902	29	31	938	A-AFFY-59	May 23, 2013
Poplar	Ppo.c1-0	21,909	53	23	404	A-AFFY-131	May 23, 2013
Grape	Vvi.c1-0	8,351	32	14	245	A-AFFY-78	May 23, 2013
Alfalfa	Mtr.c1-0	4,166	9	43	585	A-AFFY-71	May 23, 2013

Rice	Osa.c3-0	20,625	53	73	1214	A-AFFY-126	May 23, 2013
Maize	Zma.c1-0	8,397		47	617	A-AFFY-77	May 23, 2013

*^a^* Gene coverage indicates the percentage of protein-encoding genes (provided by Phytozome v9.1) that are included in the coexpression data set (Goodstein et al. 2012). Statistics for maize are not provided because of poor annotation quality.

*^b^* This column indicates the number of slides for each microarray platform and the number of runs for the RNAseq platform (Ath2).

### Overall performance for gene coexpression data

Because gene coexpression data sets can be constructed using many types of expression data and many types of methods, it is necessary to evaluate the data carefully. We previously used the predictive performance of GO annotations to evaluate coexpression data sets (Obayashi and Kinoshita 2009, Kinoshita and Obayashi 2009) because coexpressed genes probably share functional properties. Herein, we partially modified our previous assessment procedure to provide a simpler interpretation. We compared coexpression values between two sets of gene pairs; one pair shared at least one GO term, whereas the other pair did not. With the use of different coexpression thresholds, a receiver operating characteristic (ROC) curve was prepared for each coexpression data set. As a representative value of the ROC curve, we used AUC_0.01_ (the area under the ROC curve up to the point where the false-positive rate = 0.01) (McClish 1989) because, when using these gene coexpression data sets, researchers typically select highly coexpressed pairs of genes for further study. In particular, to draw coexpressed gene networks in ATTED-II, we considered only the top three connections for each gene. Nevertheless, the conventional ROC AUC value was universally reflected by the order of very weak coexpression (e.g. several hundredths or thousandths of the strongest coexpression), which is generally too weak for ordinary coexpression analyses. We therefore used AUC_0.01_ to focus on the performance of more strongly coexpressed genes.

Table 2 shows the predictive value of GO annotations for coexpression data presented in the current ATTED-II database. For comparison, predictive performance is also shown for previous versions of Arabidopsis (Ath.c4-1) and rice (Osa.c2-0) coexpression data (italicized lines). The performance using Ath.c5-0 (7.27) is superior to that when Ath.c4-1 (5.97) is used and slightly better for rice when Osa.c3-0 (3.73) instead of Osa.c2-0 (3.63) is used (GO score in Table 2). One limitation with using GO terms to perform these quality assessments is that the assessment depends on the quality of the GO terms for each species. Even for the most intensely studied plants Arabidopsis and rice, the number of selected GO terms associated with a gene can be quite different (Table 3). We therefore developed an alternative quality assessment method that uses codon usage. Previous reports indicate that codon usage is related to gene function. For example, genes with similar expression patterns (Plotkin et al. 2004, Najafabadi et al. 2009, Camiolo et al. 2012) or genes that encode interacting proteins (Najafabadi and Salavati 2008) have similar patterns of codon usage, possibly owing to varying abundance of diverse tRNAs in different tissues. Given the results of these reports, we constructed a gene similarity matrix based on codon usage. We then measured the degree of coincidence between the coexpression data and the codon usage similarity matrix. To measure similarity between these two gene lists, we previously proposed a similarity measure COXSIM that is the weighted concordance rate of the top 100 genes in the two lists (Obayashi et al. 2013). The reasoning behind this analysis is similar to why we used the partial AUC_0.01_ in that we focused on eliminating false positives. Table 2 shows the degree of coincidence between gene coexpression and codon usage similarity. As expected, the degree of coincidence was greatest for the current Arabidopsis coexpression data set (Ath.c5-0). These coincidence scores are also listed for the new species (Table 2). The score for soybean is the largest, whereas the alfalfa score is the smallest, suggesting that the alfalfa data cannot be used in the same manner as the Arabidopsis data. Given this result and the fact that alfalfa covered the smallest total number of genes, we did not include the alfalfa data (Mtr.c1-0) in the parallel view (Fig. 1). Instead, the alfalfa data are released as only a downloadable table to be used in combination with other large-scale data sets. This restriction will be removed in future updates.

**Table 2:** Development of coexpression data performance

Species	Version	No. of genes	No. of samples*^b^*	GO score*^c^*	Codon score*^d^*
Arabidopsis	Ath.c5-0	20,836	11,171	7.27	4.02
*Arabidopsis**^a^*	*Ath.c4-1*	*20,906*	*1388*	*5.97*	*2.48*
Arabidopsis	Ath2.c1-0	25,838	328	4.88	2.63
Soybean	Gma.c1-0	15,902	938		2.53
Poplar	Ppo.c1-0	21,909	404		1.77
Grape	Vvi.c1-0	8,351	245		1.42
Alfalfa	Mtr.c1-0	4,166	585		1.37

Rice	Osa.c3-0	20,625	1214	3.73	2.38
*Rice**^a^*	*Osa.c2-0*	*20,125*	*310*	*3.63*	*2.18*
Maize	Zma.c1-0	8,397	617		1.96

Random				0.5	1.00

*^a^* Italicized lines indicate previous versions of Arabidopsis and rice coexpression data.

*^b^* This column indicates the number of slides for each microarray platform and the number of runs for the RNAseq platform (Ath2).

*^c^* Predictive performance of the GO annotation represented by AUC_0.01_ (E–4). A larger score indicates a better performance.

*^d^* Coincidence score with codon similarity represented by the median of the normalized COXSIM value. A larger score indicates a better performance.

**Table 3:** Number of GO BP terms and genes to validate the predictive power of the gene coexpression data

Coexpression data	No. of GO BP terms	No. of assessed genes
Ath.c5-0	2,785	3,410
*Ath.c4-1**^a^*	*2,950*	*3,613*
Ath2.c1-0	2,950	4,058
Osa.c3-0	679	203
*Osa.c2-0**^a^*	*690*	*193*

*^a^* Italicized lines indicate previous versions of Arabidopsis and rice coexpression data.

### Performance evaluations for each guide gene

Although the evaluation approaches described above quantify the reliability of each gene coexpression data set, it is also important to assess the reliability of each guide gene. A parallel view of gene coexpression is one way to examine coexpression reliability. Analyzing multiple species can improve coexpression performance (Stuart et al. 2003, Oti et al. 2008), as gene coexpression present in multiple species, i.e. conserved coexpression, is more reliable. Given this logic, we previously defined significance levels for genes in a mammalian coexpression database (Obayashi et al. 2013).

**Fig. 2:**
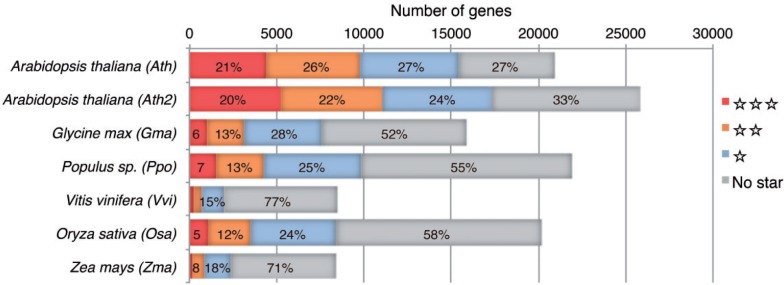
Number of genes associated with each reliability level. Reliability levels are represented by stars. Three stars indicate excellent reliability, whereas no stars indicates not reliable. The numbers within the bars indicate the percentage of each reliability category for each species. Genes with no stars include genes without orthologs.

We applied similar significance levels to ATTED-II but made modifications because orthologous relationships are more complicated in plants. Based on orthologous gene data released by the Plant Genome Database Japan, the number of orthologs associated with a particular gene is highly variable, ranging from 0 to about 100. This variability makes statistical comparisons difficult. For each gene, therefore, a BLASTP search was performed (e-value <1E–5; Altschul et al. 1997), and the top three genes were considered as candidate gene orthologs to be used to calculate the COXSIM value. After selecting the maximum COXSIM value obtained by comparing the data in seven reference platforms, then the significance of the maxCOXSIM value was determined from the null distribution of the comparisons. Note that the three candidate orthologs (identified using BLASTP) may not include the true functional ortholog, particularly in the case of a large gene family, and that the lack of support data does not directly mean the guide gene is defective. The degree of significance is indicated by stars on the gene list in ATTED-II. Single, double and triple stars correspond to *P*-values <1E–4, 1E–12 and 1E–30, respectively. Coexpression of genes with poor reliabilities can be removed using row and column filters (Fig. 1). The number of genes at each significance level is shown in Fig. 2. In general, conservation-based reliability displays a similar trend to codon usage-based reliability (Table 2), although the number of stars depends on the existence of close species. For example, maize genes typically have fewer stars because close species lack accurate coexpression data. In contrast, Arabidopsis has many more three-star genes because coexpression comparisons were mainly performed using the same species (Ath and Ath2). This notwithstanding, the high coexpression values in Arabidopsis once again provide high confidence in the reliability of coexpression targets obtained, independently of the analytical platform.

### For mashup services using coexpression data

In addition to the bulk download functions (http://atted.jp/download.shtml) and the API settings (see http://atted.jp/help/API.shtml), coexpressed gene pairs [mutual rank (MR) <100] in any species are now available in SPARQL for the semantic web communities, using the Virtuoso Universal Server at (http://atted.jp/sparql). This will promote the development of mashup applications with various omics data sets. In total, approximately 50 million triplets are provided, where a pair of gene IDs is used as the subject and the single gene ID or coexpression strength is used as the object. Sample codes to link coexpression data and UniProt data are shown on this page.

## Materials and Methods

### Construction of gene coexpression data

To generate microarray-based gene coexpression data, we downloaded GeneChip CEL files from ArrayExpress (Rustici et al. 2013). The MR value of the weighted Pearson’s correlation coefficient was used as the measure of coexpression, as described (Obayashi and Kinoshita 2009). Orthologous gene relationships were downloaded from the ortholog database in the Plant Genome Database Japan to construct the parallel view (Fig. 1).

To generate RNAseq-based gene coexpression data, we downloaded data from the Sequence Read Archive (Kodama et al. 2012) at the DNAnexus site (http://sra.dnanexus.com/). These data were converted to FASTQ format and mapped onto the mRNA sequences of Arabidopsis, using Bowtie2 (Langmead and Salzberg 2012). Low quality data (total mapped counts <5,000,000) were filtered out, leaving 328 runs that corresponded to 28 experiments. Mapped counts were summed for each gene model and used as the gene expression value. Genes with low levels of expression, i.e. their largest counts across all runs were <100, were omitted. After conversion to a base-2 logarithm with a pseudo count of 1, quantile normalization was applied to the data of each experiment, and the average expression levels were subtracted for each gene. Using all experiments at once, Pearson’s correlation coefficients for each gene pair were calculated, and these values were transferred to the MR value (Obayashi and Kinoshita 2009). Note that in this case, quantile normalization (Bullard et al. 2010) performed better for the GO test than did the following normalization methods: RPKM (Mortazavi et al. 2008), upper quartile (Bullard et al. 2010), TMM (Robinson and Oshlack 2010) and RLE (Anders and Huber 2010) (data not shown).

### Predictive performance of GO terms by gene coexpression data

Given the different importance of GO terms along with their hierarchical topologies, we selected GO terms for evaluating coexpression data as described (Kinoshita and Obayashi 2009), with slight modifications. We selected GO terms associated with 1–20 genes. Genes associated with at least one selected GO term were used in this assessment. The number of GO Biological Process terms and the number of genes used for each platform are shown in Table 3. All gene pairs in a platform were divided into two groups: those that shared at least one GO term and those that did not. The difference in the distributions of degrees of coexpression was assessed using ROC AUC_0.01_.

### Coincidence score with codon similarity

Protein-encoding sequences were retrieved from TAIR (Lamesch et al. 2012), RAP-DB (Sakai et al. 2013) and NCBI GenBank (Benson et al. 2013). For each gene, a 61-dimension vector was constructed from the number of codons in the protein-encoding sequence. Pearson’s correlation coefficients for vectors between all gene pairs were calculated and used to indicate codon usage similarity. For each guide gene, the gene list was then ordered on the basis of the strength of the codon usage similarity. Finally, the gene list was compared with the coexpressed gene list to assess the quality of the coexpression data.

### Similarity of gene lists

To measure similarity between two gene lists, we used the COXSIM value (Obayashi et al. 2013), which provides asymmetric modification of the ordered gene list proposed by Yang et al. (2006) to manage multiple gene matches between two lists of genes.

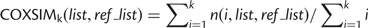

where *n*(*i*, *list*, *ref_list_*) is the number of genes in the top *i* genes of *list* that have a corresponding gene in the top *i* genes of *ref_list_*. Note that we did not count the number of gene pairs between *list* and *ref_list_* but the number of genes in *list*. Focusing on one list makes it possible to compare gene lists that include multiple gene matches. For assessment of a coexpressed gene list, we set *k* to 100, which means that we checked gene correspondence for the top 100 coexpressed genes, a reasonable limit when designing a biological experiment (Obayashi and Kinoshita 2010). To use this measure to evaluate a guide gene, we prepared a series of COXSIM values between the guide gene of interest and those in other reference platforms. Genes from other reference platforms included the same guide gene in the same species and orthologous guide genes in other species. As the representative COXSIM value of the target guide gene, we used the maximal COXSIM value (maxCOXSIM). This minimized effects of unreliable gene expression data and inaccurate gene ortholog predictions.

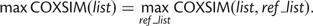



Because the expected value of maxCOXSIM depends on the total number of genes in the *list*, for the interspecies comparison in Table 2, the maxCOXSIM value was divided by its expected value. The significance of the maxCOXSIM value was also assessed using the null distribution for each platform. The degree of significance is represented by stars on the gene list in ATTED-II, where single, double and triple stars correspond to *P*-values <1E–4, 1E–12 and 1E–30, respectively.

## Funding

This research was supported by the Japan Science and Technology Corporation [CREST research project (11102558 to T.O.); and by Grants-in-Aid [for Innovative Areas (24114005 to T.O.; 22136005 to K.K.), for Scientific Research (24570176 to K.K.) and Publication of Scientific Research Results (247005 to T.O.)].

## Abbreviations

AUC: area under the curve
GO: gene ontology
MR: mutual rank
ROC: receiver operating characteristic
RNAseq: RNA sequencing
